# Does Vitamin D Deficiency Cause Hypertension? Current Evidence from Clinical Studies and Potential Mechanisms

**DOI:** 10.1155/2010/579640

**Published:** 2009-11-10

**Authors:** M. Iftekhar Ullah, Gabriel I. Uwaifo, William C. Nicholas, Christian A. Koch

**Affiliations:** ^1^Division of General Internal Medicine and Hypertension, Department of Medicine, University of Mississippi Medical Center, Jackson, MS 39216, USA; ^2^Division of Endocrinology, University of Mississippi Medical Center, Jackson, MS 39216, USA; ^3^Department of Medicine, G.V. (Sonny) Montgomery VA Medical Center, Jackson, MS 39216, USA

## Abstract

Vitamin D deficiency is widely prevalent across all ages, races, geographical regions, and socioeconomic strata. In addition to its important role in skeletal development and calcium homeostasis, several recent studies suggest its association with diabetes, hypertension, cardiovascular disease, certain types of malignancy, and immunologic dysfunction. Here, we review the current evidence regarding an association between vitamin D deficiency and hypertension in clinical and epidemiological studies. We also look into plausible biological explanations for such an association with the renin-angiotensin-aldosterone system and insulin resistance playing potential roles. Taken together, it appears that more studies in more homogeneous study populations are needed before a firm conclusion can be reached as to whether vitamin D deficiency causes or aggravates hypertension and whether vitamin D supplementation is safe and exerts cardioprotective effects. The potential problems with bias and confounding factors present in previous epidemiological studies may be overcome or minimized by well designed randomized controlled trials in the future.

## 1. Introduction

Vitamin D deficiency has been described worldwide [1]. In addition to its important role in skeletal development and calcium homeostasis, it has been suggested that low vitamin D nutritional status may have an impact on extra-skeletal health including increased risk of certain types of malignancy, immunologic dysfunction, diabetes, and cardiovascular disease [2, 3]. The socioeconomic burden of the above chronic diseases that have been linked to vitamin D deficiency is enormous. The estimated yearly total health costs for persons in the United States with heart disease and hypertension was 148 billion US dollars in 1996 [4]. Since both hypertension and vitamin D deficiency are highly prevalent worldwide, establishing an association among these two may potentially have wide public health implications but may also be the result of the high prevalence of both conditions rather than a causative link between them. Given the low cost and low side effect profile of vitamin D, replacing it in vitamin D deficient populations may help reduce the burden of this condition and simultaneously reduce cardiovascular risk.

## 2. Biological Roles of Vitamin D in Humans

There are two main sources of vitamin D available to humans, from direct exposure to sunlight (Solar UV-B) and from the diet or dietary supplements. Solar UV-B radiation penetrates the skin and converts 7-dehydrocholesterol to previtamin D3, which, in turn, converts rapidly to vitamin D3. The oily fishes such as salmon, sardines, and mackerel, egg yolks, and fish oils such as cod liver oil contain vitamin D naturally. Fortified milk, cereal, juice, and yogurt are dietary sources in North America. The cutaneous synthesis of vitamin D may vary widely in different populations depending on their availability of sun exposure as well as the actual sun exposure of the bare skin when the sun light is available. Skin pigmentation with melanin is a limiting factor in the cutaneous synthesis of vitamin D. Melanin acts as an effective natural sunscreen and, therefore, increased skin pigment can greatly reduce the solar UV-B-mediated cutaneous synthesis of vitamin D3 by as much as 99% [5].

Vitamin D from the skin and diet is metabolized in the liver to 25-hydroxyvitamin D[25(OH)D]. This 25(OH)D is metabolized in the kidneys by the enzyme 25-hydroxyvitamin D-1*α*-hydroxylase (CYP27B1) to its active form, 1,25-dihydroxyvitamin D[1,25(OH)_2_D]. The renal production of 1,25(OH)_2_D is closely regulated by serum parathyroid hormone (PTH) levels as well as by serum calcium and phosphorus levels [6]. The absorption of renal calcium as well as intestinal calcium and phosphorus is increased in the presence of 1,25(OH)_2_D [7].

The 1,25(OH)_2_D ligand binds to the vitamin D receptor (VDR) and triggers an increase in intestinal absorption of both calcium and phosphorus. Vitamin D is also involved in bone formation, resorption, and mineralization as well as in maintaining neuromuscular function. Circulating 1,25(OH)_2_D reduces the serum PTH level directly by decreasing parathyroid gland activity and indirectly by increasing serum calcium. It also regulates bone metabolism in part by interacting with the VDR in osteoblasts to release biochemical signals, leading to formation of mature osteoclasts. The osteoclasts release collagenases and hydrochloric acid to dissolve the matrix and mineral, releasing calcium into the blood [8]. 

 Decreasing vitamin D levels impairs the calcium and phosphorus homeostasis in the body. Vitamin D is primarily responsible for regulating the efficiency of intestinal calcium absorption. Its deficiency decreases the absorption of calcium from the small intestine. This leads to a cascade of events by increasing the production and release of PTH into the circulation, which restores calcium homeostasis by increasing tubular reabsorption of calcium in the kidney, increasing bone calcium mobilization from the bone, and enhancing the production of 1,25(OH)_2_D [8]. 

 Even though the bone, small intestine, and kidneys are the primary organs responsive to vitamin D, the effects of vitamin D in the body are more far reaching. The vitamin D receptor (VDR) has been identified in many cell types, tissues, and organs, including those not typically associated with calcium homeostasis and bone metabolism indicating that vitamin D may also be involved in important biological processes beyond calcium homeostasis [9]. Some of these include the heart, vascular smooth muscle, endothelium, stomach, pancreas, brain, skin, gonads, and various cells of the immune system [8]. VDR is a steroid hormone nuclear receptor that binds to 1,25(OH)_2_D with high affinity and mediates transcriptional gene regulation [8]. Recent evidence suggests that the VDR is also involved in mediating noncalcemic effects of vitamin D and its analogues and may play a vital role in disease prevention and maintenance of extra-skeletal health. A number of recent studies involving cell culture and VDR knockout mice have suggested that vitamin D plays a critical role in regulating the Renin-Angiotensin System (RAS) and thus influencing the regulation of blood pressure [10, 11].

## 3. Observations of Racial and Geographical Factors Influencing Vitamin D Nutritional Status

There are various cultural, social, racial, and geographical factors that may influence the inadequacy of vitamin D. Degree of exposure of bare skin to the sunlight is an important factor in determining vitamin D status. A change in season or latitude may have a dramatic effect on the cutaneous production of cholecalciferol. In the INTERSALT study, which examined >10 000 participants from around the world, the systolic and diastolic blood pressure were significantly and positively associated with distance from the equator [12, 13]. In Edmonton, Canada (52° N), the photosynthesis of precholecalciferol essentially ceases by mid-October and does not resume until mid-April, thus influencing the vitamin D concentration in that population [14]. Covering the entire body with clothing (as customary in women in some religions), use of sunscreen and glass shielding may significantly reduce or completely eliminate the production of vitamin D3 in the skin [15]. Biological factors that inhibit cutaneous vitamin D synthesis and bio-availability include skin pigmentation, medication use, body fat content, fat malabsorption, and age [16]. Increased skin pigmentation can reduce cutaneous vitamin D3 production as much as 99.9%, which may explain, at least in part, the higher prevalence of vitamin D deficiency in the African American population [5]. In addition, ethnic-specific polymorphisms of the vitamin D receptor gene and its promoter may influence vitamin D action including individual calcium absorption.

## 4. Epidemiological Evidence of the Relationship between Vitamin D and Hypertension

Several clinical and epidemiological studies have shown that there may be an association between hypertension and vitamin D status as well as calcium metabolism. In 1980, McCarron et al. [17] postulated that disorders of calcium metabolism including hyperparathyroidism may be related to development of hypertension and showed in his study that hypertensive patients had a significant (*P* < .005) relative hypercalciuria when compared to normotensives*. *In the early 1990s, Cooper and Rotimi observed geographic differences in blood pressure among individuals of African origin. He found that those residing in the northern regions had higher blood pressure than those residing closer to the equator [18]. Langford and Watson [19] noted that children from rural Mississippi had higher blood pressure and low calcium intake compared to urban children. Zemel et al. [20, 21] found that the intake of calcium in salt-sensitive blacks can reduce blood pressure and cause partial regression of left ventricular hypertrophy. Other investigators have found similar results [22, 23].

Scragg et al. [24] recently reported their findings of the relationship between serum 25(OH)D concentration and blood pressure. The authors studied the data from the third National Health and Nutrition Examination Survey (NHANES III), a nationally representative cross-sectional survey of the noninstitutionalized population in the United States carried out during 1988–1994. A signi*ﬁ*cant inverse association was reported between serum 25(OH)D concentration and blood pressure that was evident even after adjustment for variables including age, gender, ethnicity, and physical activity. After dividing the subjects into 25(OH)D quintile groups, the authors found that the mean systolic blood pressure was 3.0 mm Hg lower and the diastolic blood pressure was 1.6 mm Hg lower in the highest quintile [serum 25(OH)D ≥85.7 nmol/L], compared with the lowest quintile [serum 25(OH)D ≤40 nmol/L] of vitamin D status. The mean difference in systolic BP was higher among non-Hispanic blacks than Mexican Americans compared to non-Hispanic whites, after adjusting for age and sex. This difference in systolic BP in non-Hispanic blacks (compared to non-Hispanic whites) becomes blunted when quintile of vitamin D is added to the model, indicating that higher prevalence of vitamin D deficiency in non-Hispanic blacks has contributed to the higher mean systolic BP in this group of population.

Judd et al. [25] also analyzed the NHANES III survey data and showed a statistically significant inverse association between circulating 25(OH)D concentrations and systolic blood pressure. However, this association was not statistically significant when age was included in the model, nor was it significant in the black subpopulation. 

Martins et al. [26] found that a low vitamin D level was associated with a higher risk of having hypertension. This cross-sectional study, using the NHANES III data, looked at the association between serum 25(OH)D and several cardiovascular disease risk factors, including hypertension, in the adult US population. The 25(OH)D levels were found to be significantly lower in women, elderly persons (≥60 years), racial/ethnic minorities, and participants with obesity, hypertension, and diabetes mellitus. The adjusted prevalence of hypertension in adults in the US was 30% higher in the lowest quartile compared to the highest quartile of serum 25(OH)D. 

In summary, the NHANES III data analyses showed an inverse association between blood pressure and vitamin D concentration, even though it is not consistent across all different groups of subpopulations in the above three analyses. This is most likely due to the differences in the variable adjusted and the samples included in the analyses.

Forman et al. [27] prospectively investigated the independent association between plasma 25(OH)D levels and risk of incident hypertension. Two prospective cohort studies that included 613 men from the Health Professionals' Follow-Up Study and 1 198 women from the Nurses' Health Study with measured 25(OH)D levels were followed for 4 to 8 years. After this follow-up, the multivariable relative risk (RR) of incident hypertension among those whose measured plasma 25(OH)D levels were <15 ng/mL (compared with those whose levels were >30 ng/mL) was 6.13 in men and 2.67 in women. These findings provide support in favor of an association between vitamin D deficiency and the increased risk of hypertension.

## 5. Clinical Studies Showing Effect of Vitamin D Supplementation on Blood Pressure

Increasing vitamin D level in the blood directly or indirectly has been shown to reduce blood pressure in some studies. Krause et al. randomly assigned 18 patients with mild hypertension to receive UV-B or UV-A exposure, 3 times weekly for 6 weeks [28]. In this study, he found that there was a 162% rise in plasma 25(OH)D in the UV-B group along with a drop in both systolic and diastolic blood pressure by 6 mm Hg. No change in the blood pressure was observed with UV-A exposure (UV-A does not produce vitamin D). In another randomized, placebo-controlled study in 145 elderly women showed that 800 IU of vitamin D3 plus 1200 mg of calcium significantly reduced blood pressure by 9.3% after 8 weeks, whereas treatment with 1200 mg of calcium alone reduced blood pressure by only 4.0% (*P* = .02) [29].

## 6. Epidemiological Studies Showing No Relationship between Vitamin D and Hypertension

Even though the majority of recent clinical studies cited earlier in this review support the hypothesis of an inverse association between the vitamin D serum level and blood pressure, there have been studies that contradict this hypothesis as well. For instance, a large prospective study by Forman et al. in 2005 found no association between vitamin D intake from diet and supplements and the risk of incident hypertension [30].

In the Women's Health Initiative Calcium/Vitamin D Trial by Margolis et al. supplementation of 1000 mg of elemental calcium plus 400 IU of vitamin D3 daily (versus placebo) in a random double-blind fashion did not show any significant decrease in incidence hypertension after a median followup time of 7 years [31]. In another longitudinal, placebo-controlled, double-blind study by Orwoll Oviatt, normotensive men were treated with a calcium and cholecalciferol supplement, or placebo, for 3 years without any demonstrable effect on systolic, diastolic, or mean arterial pressure [32]. The randomized double-blind trial by Scragg et al. with a vitamin D supplementation at a single dose of 2.5 mg in winter months did not show any significant decrease in blood pressure after 5 weeks when compared to placebo [33]. Snijder et al. studied the participants of the Longitudinal Aging Study in Amsterdam and found that blood pressure in this population was not inversely associated with the serum 25(OH)D level but was positively correlated with serum PTH [34]. Another study conducted in Germany showed similar findings [35].

## 7. Possible Cause/Explanation for Lack of an Association between the Vitamin D Level and Blood Pressure

The reasons behind these contradictory findings in the above mentioned studies about an association of blood pressure and vitamin D could be multiple. The earlier observational study by Forman did not have information about the exact amount of vitamin D intake and it was estimated from a semiquantitative food frequency questionnaire [30]. The diagnosis of hypertension was also only self-reported in this study, not by direct measurement of the participant's blood pressure. Unless the subjects with lower intake of vitamin D had clinically significant deficiency, an association between vitamin D status and blood pressure is likely to be missed. If vitamin D decreased blood pressure mainly via inhibiting the RAS, we may not see any decrease in blood pressure if the subjects do not have a vitamin D level below a certain threshold, at which a hyperreninemic state is created. Thus, vitamin D supplementation would also be unlikely to produce any decrease in blood pressure in normotensive subjects as their plasma renin is likely to be normal. The study by Scragg et al. measured blood pressure response only after 5 weeks of vitamin D intake which may be too early to show any clinical response [33]. In the study by Snijder et al., the authors postulated that the lack of an association between blood pressure and vitamin D level may be due to the relatively high levels of vitamin D in that population [34].

The more recent randomized controlled trial by Margolis et al. [31] was a substudy of the Women's Health Initiative (WHI) that was originally designed to look at hip fractures and colorectal cancers. In an editorial comment about this study, Geleijnse mentioned that there may be several flaws in this study [36]. The level of compliance to vitamin D/calcium was only modest (60% women reported taking >80% study medications); BP measurement criteria were not strict at the site visit and were probably not blinded to staff and participants. Nearly half of the participants were already hypertensive at baseline and 28% used antihypertensive medications. Incident hypertension was self reported, based on asking women twice per year if they had been started on new “pills for hypertension.” The vitamin D dose used in the study (400 IU) was fairly low, and the estimated increase of the vitamin D level with this supplementation in this study population was about 2 ng/mL (5 nmol/L) [37] which may be too low to produce any clinical effect on blood pressure. In the study by Judd et al., [25] the authors suspected that using a 25(OH)D cutoff point of <80 nmol/L to define vitamin D insufficiency had placed the majority of blacks (92%) into the vitamin deficient group, leaving too few people in the nondeficient group, resulting in failure to detect a statistically significant association between hypertension and vitamin D. The inverse association between blood pressure and 25(OH)D concentration was further weakened after controlling for age because vitamin D production decreases in older adults secondary to lower skin concentrations of 7-dehydrocholesterol (the precursor to D_3_). Increasing age is also associated with decreased concentrations of 25(OH)D [38]. Since these two variables are highly correlated, adjusting for one (age, in this case) had weakened the association of the other variable (vitamin D level) with systolic blood pressure in this study.

## 8. Evidence of a Relationship between Vitamin D and Preeclampsia

Maternal vitamin D deficiency is a widespread public health problem. In a recent study, it was found that approximately 29% of black pregnant women and 5% of white pregnant women residing in the northeastern United States had vitamin D deficiency (25(OH)D <37.5 nmol/L), whereas 54% of black women and 47% of white women had vitamin D insufficiency with a serum 25(OH)D levels of 37.5–80 nmol/L [39]. Preeclampsia is a pregnancy-specific syndrome that affects approximately 3%–7% of first pregnancies. The known racial disparity in preeclampsia, with black women being more likely to develop severe preeclampsia and suffer greater morbidity associated with the disorder than white women [40], is consistent with the hypothesis that vitamin D deficiency may be involved in the development of this condition. 

Hyppönen et al. [41] investigated the association between infant vitamin D supplementation and development of preeclampsia in those persons later in life when they become pregnant. The investigators used data on 2 969 women born in the Northern Finland Birth Cohort 1966 of whom 68 (2.3%) had preeclampsia in their first pregnancy. Risk of preeclampsia was halved ((OR) 0.49, 95% CI 0.26–0.92) in participants who had received vitamin D supplementation regularly during the first year of life. Studies of seasonal patterns in preeclampsia showed that the incidence of preeclampsia was the lowest in summer, when sunlight is plentiful and serum 25(OH)D concentrations are at their peak, and the highest in winter, when synthesis of vitamin D3 is limited in temperate zones and serum 25(OH)D levels are at their nadir [42, 43]. In a nested case-control study by Bodnar et al. [44], pregnant women were followed from less than 16-week gestation to delivery at prenatal clinics and private practices. Patients included nulliparous pregnant women with singleton pregnancies only. Adjusted serum 25(OH)D concentrations in early pregnancy were 15% lower in women who subsequently developed preeclampsia compared to controls. Early-pregnancy maternal 25(OH)D concentration less than 37.5 nmol/L was associated with a 5-fold increase in the odds of preeclampsia, independent of race/ethnicity, season, gestational age, prepregnancy BMI, and education. After confounder adjustment, a 50-nmol/L decline in 25(OH)D concentration doubled the risk of preeclampsia. Findings of this study indicate that maternal vitamin D deficiency may be an independent risk factor for preeclampsia. 

Very recently, Bills et al. reported that low plasma vascular endothelial growth factor (VEGF) in the first trimester is a predictive marker for preeclampsia [45]. On the other hand, Cardus et al. found that 1,25 (OH)2 vitamin D regulates VEGF production through a vitamin D response element in the VEGF promoter [46]. Thus, lack of vitamin D may have a role in pathogenesis of developing hypertension via decreased VEGF production.

## 9. Vitamin D Deficiency and Hypertension: Potential Biological Mechanisms

### 9.1. Regulation of the Renin Angiotensin System (RAS)

**Figure 1:**
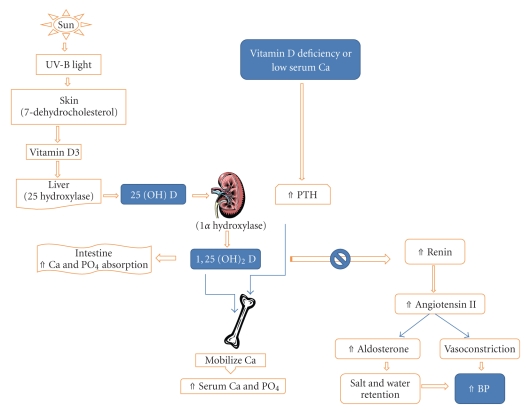
Pathway of vitamin D metabolism and its relationship with PTH and renin-angiotensin-aldosterone system.

The renin angiotensin system (RAS) is a regulatory cascade that plays a critical role in the regulation of blood pressure, electrolyte, and plasma volume homeostasis. Inappropriate stimulation of the RAS has been associated with hypertension. Li et al. [10] demonstrated that vitamin D is a potent endocrine suppressor of renin biosynthesis to regulate the RAS. Mice lacking vitamin D receptor (VDR) have elevated production of renin and angiotensin II, leading to hypertension, cardiac hypertrophy, and increased water intake. These abnormalities can be prevented by treatment with an ACE inhibitor or AT_1_ receptor antagonist. Vitamin D suppression of renin expression is independent of calcium metabolism, the volume and salt-sensing mechanisms, and the angiotensin II feedback regulation. In normal mice, vitamin D deficiency stimulates renin expression, whereas injection of 1,25-dihydroxyvitamin D_3_[1,25(OH)_2_D_3_] reduces renin synthesis. In cell cultures, 1,25(OH)_2_D directly suppresses renin gene transcription by a VDR-dependent mechanism. Thus, vitamin D-deficiency may increase the risk of hypertension, and vitamin D supplementation may be beneficial to the cardiovascular system. In a transgenic mouse model with mice over-expressing the human vitamin D receptor in renin-producing cells, Kong et al. demonstrated that suppression of renin expression by 1,25(OH)_2_ D in vivo is independent of parathyroid hormone and calcium [47] (see Figure 1).

### 9.2. Relationship of Parathyroid Hormone with Hypertension in Primary and Secondary Hyperparathyroidism

Vitamin D deficiency is prevalent in patients with primary hyperparathyroidism (HPT) [48–50]. Primary HPT with an inappropriately elevated PTH level has been shown to be associated with hypertension (in up to 40% of cases) but the mechanism of developing hypertension has remained controversial. Low vitamin D status is associated with secondary elevation of PTH as well as increased arterial resistance leading to hypertension [51]. It has also been implicated directly and indirectly to affect cardiovascular risk by increasing susceptibility to arterial calcification [52]. The patients with mild primary HPT have been found to have increased stiffness measured in the radial artery [53]. In both primary and secondary hyperparathyroidism, elevated PTH levels may contribute to elevation of blood pressure. 

Puepet et al. [54] reported a 47-year-old Nigerian man with severe hypertension (BP 210/130 mm Hg) and primary hyperparathyroidism who had a normal blood pressure after parathyroidectomy. Gennari et al. [55] studied patients with primary hyperparathyroidism with parathyroid adenoma and observed that plasma renin activity, and plasma aldosterone levels were higher among these patients who were hypertensive and the blood pressure, plasma renin activity and plasma aldosterone levels became normal after parathyroidectomy in most of them. These results are consistent with the hypothesis of a possible direct effect of PTH on renin secretion, which could contribute to the pathogenesis of hypertension. However, there are contradictory results as well. Bollerslev et al. studied the effect of parathyroidectomy on cardiovascular risk factors in patients with mild primary hyperparathyroidism and found no significant differences between the groups for blood pressure or markers of insulin resistance [56]. On the other hand, Rydberg et al. [57] found increased blood pressure values after parathyroidectomy in patients with primary hyperparathyroidism and a history of hypertension. Jorde et al. found that serum PTH was a significant predictor of rise in systolic blood pressure over a period of 7 years in men (*P* < .01), but not in women [58]. Fardella and Rodriguez-Portales observed a positive correlation between PTH and intracellular calcium and suggested that PTH may act as an ionophore for calcium entry into cells and perhaps react a prehypertensive condition in normotensive patients with primary hyperparathyroidism [59].

### 9.3. Vitamin D Deficiency, Obesity, and Insulin Resistance

An association of obesity with low serum vitamin D levels has been reported. Physical inactivity including decreased outdoor activities may lead to diminished exposure to ultraviolet light. This may partly account for the lower level of serum vitamin D in overweight and obese participants, who are more likely to be sedentary in their lifestyle. Analyzing the NHANES III data, Scragg and Camargo Jr. reported a 25 percent reduction in the prevalence in the vitamin D deficiency among the study population who had increased outdoor physical activities in the previous month prior to data collection [60]. In addition, the lipid solubility of vitamin D also modifies its bioavailability due to sequestration in the adipose tissue and may contribute to the lower serum levels of vitamin D in overweight and obese persons [61]. There are some observational and case-control studies suggesting that hypovitaminosis D is associated with decreased insulin secretion [62] and that vitamin D supplementation reduces the concentrations of free fatty acids in diabetics, thereby improving insulin sensitivity [63]. There is a high prevalence of type 2 diabetes and insulin resistance in patients with primary hyperparathyroidism which could aggravate hypertension and inflammation, further contributing to the cardiovascular risk in these patients [64, 65]. In a prospective study by Ahlström, PTH correlated with several metabolic factors within a normocalcemic study population and individuals with mild primary HPT had significantly more NCEP criteria for the metabolic syndrome [66].

Scragg et al. reported hypovitaminosis D being associated with higher insulin resistance and increased risk of diabetes [67, 68]. Pittas et al. prospectively evaluated the effect of vitamin D supplementation (400 IU per day) on fasting glucose which improved after a 3-year period of therapy when compared to subjects on placebo [69]. In a Framingham Offspring study involving nondiabetic individuals, a strong inverse correlation between serum vitamin D concentrations and fasting plasma glucose as well as fasting insulin levels was found, after adjustment for age, gender, BMI, waist circumference, and smoking [70]. In another cross-sectional study involving hemodialysis patients [71], low serum vitamin D was independently associated with diabetes mellitus, higher brain natriuretic peptide levels, higher pulse pressure, and higher vascular calcification scores.

**Figure 2:**
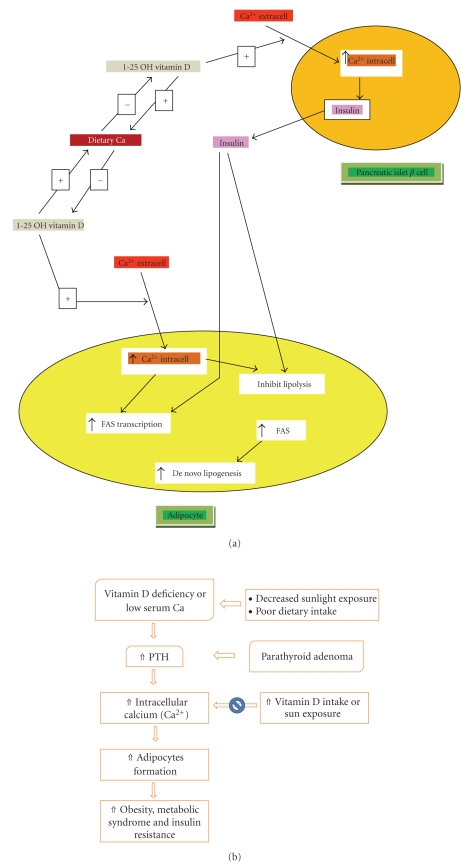
(a) Dietary and intracellular calcium + vitamin D modulation of adiposity. (b) Complex relationship between vitamin D, parathyroid hormone, intracellular calcium, and adipocyte formation.

Resnick and colleagues espouse an “ionic”-based theory for the onset and development of hypertension, type 2 diabetes, obesity, and other manifestations of the metabolic syndrome that is characterized by elevated intracellular calcium, reduced intracellular magnesium, and reduced intracellular pH [72–76]. Intracellular calcium appears to have a biphasic effect on the differentiation of preadipocytes to adipocytes. Low serum calcium resulting from low dietary intake or vitamin D deficiency leads to secondary elevation of PTH which, in turn, causes increased intracellular calcium [77] that leads to increased differentiation of preadipocytes to adipocytes [78]. It also inhibits GLUT-4 function impeding insulin mediated glucose uptake [79]. On the other hand, increased dietary calcium appears to be associated with inhibition of serum 1,25 hydroxy vitamin D which results in decreased intracellular calcium levels and thus decreased adipogenesis [80, 81]. There is also accumulating evidence suggesting that increased intracellular calcium has stimulating effect on adipocyte 11-beta hydroxysteroid dehydrogenase (HSD) type 1 activity similar to angiotensin 2, leading to increased cortisol production in adipocytes [82]. Among other means, this appears to be a membrane-mediated (rather than nuclear receptor mediated) effect of 1,25 hydroxy vitamin D which can be inhibited by an angiotensin receptor blocker (see Figures 2(a), 2(b)).

### 9.4. Vitamin D Deficiency, Calcium, and Endothelial Function

Hypovitaminosis D has been shown to have direct effects on the vasculature causing increased vascular resistance [83]. It is also independently associated with increased carotid intima-media thickness [84] and decreased arterial compliance [85]. Sugden et al. found that endothelial function (as assessed by flow-mediated dilation) and blood pressure improved in diabetics who ingested a single dose of vitamin D (100 000 IU) [86]. Ekmekci et al. [87] reported that elevated serum calcium and parathyroid hormone levels were independent predictors of impaired endothelial function and endothelial nitric oxide polymorphism (eNOS), which is often associated with coronary artery disease, and hypertension, did not appear to have modifying effect on the endothelial function in his study.

## 10. Conclusions

Evidence from clinical and epidemiological studies support a possible relationship between low vitamin D level and hypertension, and there are some plausible biological mechanisms as well. However, epidemiological studies are always vulnerable to multiple confounding factors that cannot be always controlled. The unique and complex interactions between hypovitaminosis D, parathyroid hormone, and calcium (both in the serum and in intracellular compartments) make it especially difficult to tease apart how much of these effects are truly unique and distinct to vitamin D. Moreover, statistically significant associations between two factors do not prove that one has been the causative factor for the other, as these two factors may be closely related to a third factor. In this case, a low vitamin D level may merely be a surrogate for the lack of outdoor physical activity. Outdoor physical inactivity itself may precipitate hypertension as well as leading to a cascade of events (including low sun-exposure and vitamin D deficiency, obesity, metabolic syndrome, and increased insulin resistance), all of which may cause or aggravate hypertension. The problems with bias and confounding factors may be overcome or minimized by well-designed randomized controlled trials. 

Results of recent interventional studies that investigated the potential benefit of vitamin D supplementation on blood pressure have not been promising. Further studies are needed to find out if and when vitamin D supplementation should be used for treating patients with hypertension. It is apparent that vitamin D supplementation may be appropriate for populations that are most vulnerable to hypovitaminosis D. But it is not clear what degree of vitamin D deficiency may activate the renin-angiotensin system (RAS) and trigger an increase in blood pressure. Most recent studies investigating the association of health conditions related to vitamin D suggest that a desirable 25(OH)D concentration may be at least 30 ng/mL (75 nmol/L) (but less than 100 ng/mL) for optimum health; yet no consensus has been reached about its cut-off level [88–90]. This already represents a limiting factor in future studies, as each individual likely has a different cut-off, depending on ethnic group and/or polymorphisms in the vitamin D receptor and its promoter which mediates vitamin D action. Therefore, more comprehensive studies may be needed including genetic profiling of study subjects with various levels of serum vitamin D to see what is the most likely cutoff level that triggers clinical hypertension. The challenge remains that hypertension is multifactorial and some individuals with other comorbidities (like smoking, obesity, physical inactivity, metabolic syndrome) may have a lower threshold for vitamin D deficiency induced clinical hypertension compared to those who donot have these risk factors. 

Because of the increasing evidence in favor of an association between vitamin D deficiency and cardiovascular diseases, it may be prudent to screen and correct hypovitaminosis D in high-risk patients such as those with resistant hypertension, nursing home residents, osteoporotic individuals, pregnant women in areas where the incidence of toxemia of pregnancy is high (south-east Asia), African Americans, and the female population in some geographic/religious groups where covering the entire body with clothing is customary. If screening is not available, supplementation with vitamin D 1000–2000 IU daily among this subpopulation may be safe and appropriate [91–93]. This can easily be achieved as vitamin D is available over the counter and is unlikely to cause any toxicity at this dosage. Finally, studies are underway to find vitamin D analogs with minimal calcemic potential but greater activity on RAS modulation. This may open a new horizon for a group of therapeutic inhibitors of the RAS and potentially offer a new class of antihypertensive drugs that may be used in hypertensive individuals with or without vitamin D deficiency.
